# The Mitigating Effect of *Citrullus colocynthis* (L.) Fruit Extract against Genotoxicity Induced by Cyclophosphamide in Mice Bone Marrow Cells

**DOI:** 10.1155/2013/980480

**Published:** 2013-11-07

**Authors:** Mohammad Shokrzadeh, Aroona Chabra, Farshad Naghshvar, Amirhossein Ahmadi

**Affiliations:** ^1^Pharmaceutical Sciences Research Center, Faculty of Pharmacy, Mazandaran University of Medical Sciences, 18 Kilometer of Farah Abad Road, Sari, Iran; ^2^Department of Toxicology and Pharmacology, Faculty of Pharmacy, Mazandaran University of Medical Sciences, Sari 48175-861, Iran; ^3^Student Research Committee, Faculty of Pharmacy, Mazandaran University of Medical Sciences, Sari 48175-861, Iran; ^4^Department of Pathology, Faculty of Medicine, Mazandaran University of Medical Sciences, Sari 48175-861, Iran

## Abstract

Possible genoprotective effect of *Citrullus colocynthis* (L.) (CCT) fruits extract against cyclophosphamide- (CP-)induced DNA damage in mice bone marrow cells was evaluated using micronucleus assay, as an index of induced chromosomal damage. Mice were preadministered with different doses of CCT via intraperitoneal injection for 7 consecutive days followed by injection with CP (70 mg/kg b.w.) 1 hr after the last injection of CCT. After 24 hr, mice were scarified to evaluate the frequency of micronucleated polychromatic erythrocytes (MnPCEs). In addition, the number of polychromatic erythrocytes (PCEs) among 1000 normochromatic erythrocytes (NCEs) per animal was recorded to evaluate bone marrow. Pretreatment with CCT significantly reduced the number of MnPCEs induced by CP in bone marrow cells (*P* < 0.0001). At 200 mg/kg, CCT had a maximum chemoprotective effect and reduced the number of MnPCEs by 6.37-fold and completely normalized the mitotic activity. CCT also led to marked proliferation and hypercellularity of immature myeloid elements after mice were treated with CP and mitigated the bone marrow suppression. Our study revealed that CCT has an antigenotoxic effect against CP-induced oxidative DNA damage in mice. Therefore, it could be used concomitantly as a supplement to protect people undergoing chemotherapy.

## 1. Introduction

Conventional cancer treatments have many modalities, all directed at killing tumor cells or preventing their proliferation. Cyclophosphamide (CP) is an alkylating agent and the most commonly used anticancer and chemotherapeutic drug. Its cytotoxic effects are the result of chemically reactive metabolites that alkylate DNA and protein, by producing cross-links [1]. Immunosuppression and normal tissue injury are the major limitations of chemotherapy [2], which give rise to numerous side effects [3, 4]. It has been reported that oxidative stress mediated disruption of redox balance after CP exposure generates biochemical and physiological disruptances. CP is a well-known mutagen and clastogenic agent [5] and produces the highly active carbonium ion, which reacts with the electron-rich area of nucleic acids and proteins [6]. CP is widely used as a genotoxic agent because it and its metabolites can bind DNA, causing damage that may result in chromosome breaks, micronucleus (Mn) formation, and cell death [6, 7].

Several studies suggest that antioxidant supplementation can influence the response to chemotherapy as well as the development of adverse side effects that result from treatment with antineoplastic agents [8]. Compounds that could reduce these side effects, as well as stimulate immunity, will be of great help in improving cancer treatment strategies. Recently there is an increasing interest in the search of potential compounds of plant origin that are capable of minimizing the toxicity induced by chemotherapy to normal cells without compromising its antineoplastic activity [9]. Natural products exerted protective effects against genotoxicity induced by CP in bone marrow cells of mice when these compounds were administrated prior to CP treatment. Antioxidant activity is the proposed mechanism for the chemoprotective effects of these natural products [10, 11]. We previously reported that hesperidin, a citrus bioflavonoid, may have antioxidative activity and can reduce the genotoxicity induced by CP in mouse bone marrow cells by decreasing micronucleus formation [10]. In addition, an antioxidative herbal medicine with high amount of flavonoids and phenolic compounds had a potent chemoprotective effect against CP-induced oxidative stress and DNA damage in mice bone marrow cells [11]. Therefore, plants with antioxidant activity can be a good source in this regard.

*Citrullus colocynthis *(L.) (CCT) is one of the native plants of the Middle East countries used in traditional medicine. CCT Schrad., Cucurbitaceae (colocynth or wild-gourd or bitter-apple), is a nonhardy, herbaceous perennial vine, branched from the base. In south-eastern of Iran, CCT locally known as Abujahl watermelon is a well recognized plant in the traditional medicine and was used by people in rural areas as a purgative, antidiabetic, and insecticide. Moreover, traditional medicine in Iran has for centuries used the fruits of CCT for the treatment of diabetes and hemorrhoids [12]. CCT contains active substances such as saponins, alkaloids, and glycosides, and it is used as antidiabetic and antioxidant [13, 14]. The aqueous extract of the CCT can ameliorate the toxic effect of streptozotocin (STZ) in the liver [15]. The plant extract may be acting as an antioxidant, which clears the reactive oxygen species (ROS) released by STZ since it has been shown that STZ induces ROS and other cytokines [16].

Because CCT has antioxidative properties, there is a likely possibility that CCT would protect against CP-induced DNA damage. Therefore, this study was undertaken to assess the effects of CCT against the genotoxicity induced by CP in mice bone marrow cells using the Mn test. In addition, the biochemical alterations characteristic of CP-induced oxidative stress markers have been measured by use of standard techniques. Histological examination of bone marrow was also used for observation of possible mitigating effect of CCT extract against myelosuppressive effect of CP and bone marrow suppression.

## 2. Materials and Methods

### 2.1. Plant Material

The fruits of CCT were collected from a local grocery store of Sari city, northern Iran. The plant was identified, authenticated by Dr. Bahman Eslami, assistant professor of plant systematics and ecology, Department of Biology, Islamic Azad University, Branch of Ghaemshahr, Iran.

### 2.2. Preparation of Extracts

The fruits were dried and the pulps were separated from the seeds. The pulps with peel were pulverized and then powdered in grinding mill. 110 g of powder was macerated in 1000 mL of ethanol (70%) for 72 hours. Following filtration by sterile gauze, the extract was dried after evaporating the ethanol under vacuum at a temperature of 40°C. The remained water extract was dried at oven temperature of 50°C. In doing so, 10 g of hydroalcoholic dried extract powder was obtained.

### 2.3. Animals

The protocol for the study was approved by the Research Committee of Mazandaran University of Medical Sciences, Sari, Iran. Male Naval Medical Research Institute (NMRI) mice weighing 22 ± 3 g were obtained from the Pasteur Institute of Iran (Amol). Before any experience, all animals were maintained 7 days under the same laboratory conditions of temperature (22°C ± 3°C), relative humidity (55% ± 5), and a 12 : 12 hours light/dark cycle and received a nutritionally standard diet and tap water *ad libitum*. The “Care and Use of Laboratory Animals” was prepared by Mazandaran University of Medical Sciences.

### 2.4. Chemicals

CP (Endoxan) was obtained from Baxter Oncology GMBH (Westfalen, Germany). 1,1-Diphenyl-2-picryl hydrazyl radical (DPPH) was purchased from Sigma Chemicals Co. (St. Louis, MO, USA). Butylated hydroxytoluene (BHT) was purchased from Merck (Darmstadt, Germany). Bovine serum albumin and a kit for protein measurement were purchased from Ziest Chem Co. (Tehran, Iran). All other chemicals were either at or purer than analytical grade.

### 2.5. Measurement of Free Radical-Scavenging Activity of CCT

The free radical-scavenging capacity of CCT was determined by bleaching of the stable DPPH [17, 18]. Different concentrations of the CCT fruits (0.5 to 3 mg/mL) were added, at an equal volume, to a methanol solution of DPPH (100 *μ*M). After 15 min at dark room temperature, the absorbance was recorded at 517 nm. IC_50_ values denote the concentration of the sample that is required to scavenge 50% of DPPH free radicals.

### 2.6. Experimental Treatment

For the Mn assay, animals were divided into seven groups (Groups 1–7, *n* = 5 for each group), which were comprised of the following: Group 1 (negative control), mice received distilled water (10 ml/kg b.w.) via intraperitoneal (i.p.) injection for 7 days. Group 2 (positive control), mice received a single genotoxic dose of CP (70 mg/kg b.w, i.p.) in distilled water (10 mL/kg b.w). Group 3–6, mice were treated with different doses of CCT (10, 50, 100, or 200 mg/kg b.w. by i.p. injection) in distilled water (10 mL/kg b.w) per day for 7 days followed by a single i.p. dose of CP 1 h after the last dose of CCT. Group 7, mice were treated with only high dose of CCT (200 mg/kg b.w. by i.p. injection) in distilled water (10 ml/kg b.w) per day for 7 days.

### 2.7. Mn Assay

The Mn test was performed as previously described [10, 11]. The bone marrow Mn test is a well-known in vivo assay for the assessment of genotoxicity and DNA damage in animals such as mice and rats. The number of MnPCEs is increased in rodent bone marrow cells exposed to chemical hazards and chromosome-breaking agents. A Mn is round with a diameter of approximately 1/20th to 1/5th of an erythrocyte. The ratio of PCE to NCE in bone marrow preparations is useful in estimating any perturbations in hematopoiesis as a result of treatment in exposed animals [19, 20].

The femora from the animals were used for estimation of Mn frequency and mitotic activity. Mice were sacrificed by cervical dislocation 24 h after CP injection. The bone marrow of both femurs was removed in the form of a fine suspension into a centrifuge tube with fetal calf serum (FCS). The cells were dispersed by gentle pipetting and collected by centrifuge at 1500 rpm for 10 min. The cell pellet was resuspended in a drop of FCS, and smears were prepared. The slides were coded to avoid any observed bias. After 48 h of air drying, smears were stained with May-Grunwald/Giemsa. For each experimental point, five mice were used, and 5000 PCEs were blindly scored microscopically to determine the percentage of MnPCE. In addition, the number of PCEs among 1000 NCEs per animal was recorded to evaluate bone marrow suppression; mitotic activity was calculated as % PCE = [PCE/(PCE + NCE)] × 100 as a direct index of cell division.

### 2.8. Measurement of Oxidative Stress Markers

To study the effect of CCT extract on oxidative bone marrow damage induced by CP, mice were pretreated with 200 mg/kg of CCT solution for 7 days followed by injection of CP (70 mg/kg b.w.) 1 hr after the last injection of CCT. Femur bone-marrow cells were collected from the mice killed by cervical dislocation 24 hr after CP treatment to estimate lipid peroxidation (MDA), reduced glutathione (GSH), and oxidized glutathione (GSSG) levels.

#### 2.8.1. Determination of Protein Content

The protein content was determined by the method described by Bradford using bovine serum albumin as the standard [21].

#### 2.8.2. Lipid Peroxidation Level

Malondialdehyde (MDA) generated by lipid peroxidation was quantified according to the method of Ohkawa et al. [22], based on thiobarbituric acid reactivity. The MDA concentration in the samples was calculated from the standard curve with 1,1,3,3-tetramethoxypropane as the standard and expressed as *μ*mol/g protein.

#### 2.8.3. Reduced and Oxidized Glutathione Levels

GSH was assayed with DTNB according to the protocol described by Ellman [23]. GSSG was assayed with DTNB, glutathione reductase, and NADPH as described previously [24]. The concentrations of GSH and GSSG were calculated from standard curves that were obtained from freshly prepared standard solutions of GSH and GSSG, respectively, and expressed as *μ*mol/g protein. The value obtained for GSH was divided by the GSSG value to get the GSH/GSSG ratio.

### 2.9. Histology of Bone Marrow

For histological examination of myeloid hyperplasia in bone marrow, mice were pretreated with 100 and 200 mg/kg of CCT solutions for 7 days followed by injection of CP (70 mg/kg b.w.) 1 hr after the last injection of CCT. Both femurs were removed from the mice killed by cervical dislocation at 24 hr after CP administration. Femurs were immersed in 10% formalin; bones were decalcified and processed with a microtome at micron slides. Routine H&E staining was performed on 6 *μ*m paraffin sections, and the slides were evaluated under light microscope. Slides were viewed and photographed using a camera microscope at 400x magnification. 

### 2.10. Statistical Analysis

Data are presented as the mean ± SD. One-way analysis of variance and Tukey's honestly significant difference (HSD) test were used for multiple comparisons of data. A *P* value less than 0.05 was considered to be significant. IC_50_ values were calculated from linear regression analyses. All measurements were replicated three times.

## 3. Results

### 3.1. Antioxidant Activity of Extract

**Figure 1:**
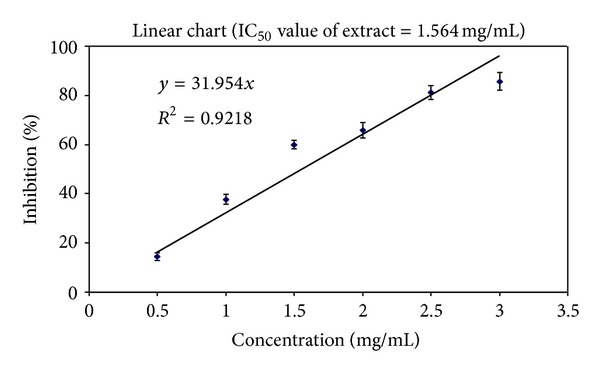
Inhibition effect of CCT fruits extract on the DPPH free radical at 517 nm.

The scavenging effect of CCT fruit extract was enhanced with increasing concentration. The maximum inhibitory effects were obtained in 95.862% at a concentration of 3 mg/ml for it. The IC_50_ value of the extract for DPPH radical-scavenging activity was 1.564 mg/mL (Figure 1).

### 3.2. Bone Marrow Mn Assay

The effect of various doses of CCT fruits extract on the frequency of MnPCEs in bone marrow cells 24 hr after CP injection is shown in Table 1. The frequency of micronuclei was significantly increased in the mice treated with CP compared with the control group (*P* < 0.0001). In mice pretreated with the CCT at the doses of 100 and 200 mg/kg, the frequency of MnPCEs induced by CP was significantly decreased compared with those treated with only CP (*P* < 0.0001). The frequency of MnPCEs was lower in the CCT + CP groups by factors of 5.9 and 6.67 for the doses of 100 and 200 mg/kg, respectively, compared with the CP treated group. The data showed that CCT suppresses the action of CP on clastogenic effects. Moreover, the mitotic activity (% PCE) in CP treated mice showed a pronounced cytotoxic effect of CP on bone marrow proliferation, and this was significantly reduced in mice bone marrow after CP treatment (*P* < 0.0001). Treatment of mice with CCT arrested the CP-induced decline in the mitotic activity (% PCE) (Table 1). CCT treatment at the doses of 100 and 200 mg/kg completely prevented the cytotoxicity induced by CP in the mice bone marrow and increased mitotic activity (% PCE) as a result of increasing bone marrow proliferation (*P* < 0.0001). CCT extract did not show any significant protective effect at a dose of 10 mg/kg. CCT alone did not also cause any cytotoxicity and genotoxicity in bone marrow cells at a high dose of 200 mg/kg.

### 3.3. Bone Marrow Lipid Peroxidation Level

The results of the analysis of bone marrow lipid peroxidation are shown in Table 2. The MDA content in mice treated with CP was significantly increased compared with the control group (*P* < 0.0001). CCT pretreatment significantly inhibited the elevation in MDA formation by CP and restored to the level of control group.

### 3.4. Bone Marrow GSSG and GSH Levels

The GSH level observed in CP-treated animals was significantly decreased, together with an increase in GSSG as compared with the control group (*P* < 0.0001) (Table 2). The GSH/GSSG ratio also decreased from 4.03 ± 1.07 to 0.28 ± 0.05, indicating increased oxidative stress. Animals pretreated with CCT showed a significant increase in GSH level over the CP-treated group to the level significantly different from the level of GSH after treatment with CP alone (*P* < 0.0001). The GSSG level was also significantly decreased in animals pretreated with CCT compared with the CP-treated group (*P* < 0.001). Consequently, the GSH/GSSG ratio was increased in CCT pretreated animals and was statistically significantly higher when compared with the CP-treated group (*P* < 0.001).

### 3.5. Histology of Bone Marrow

**Figure 2:**
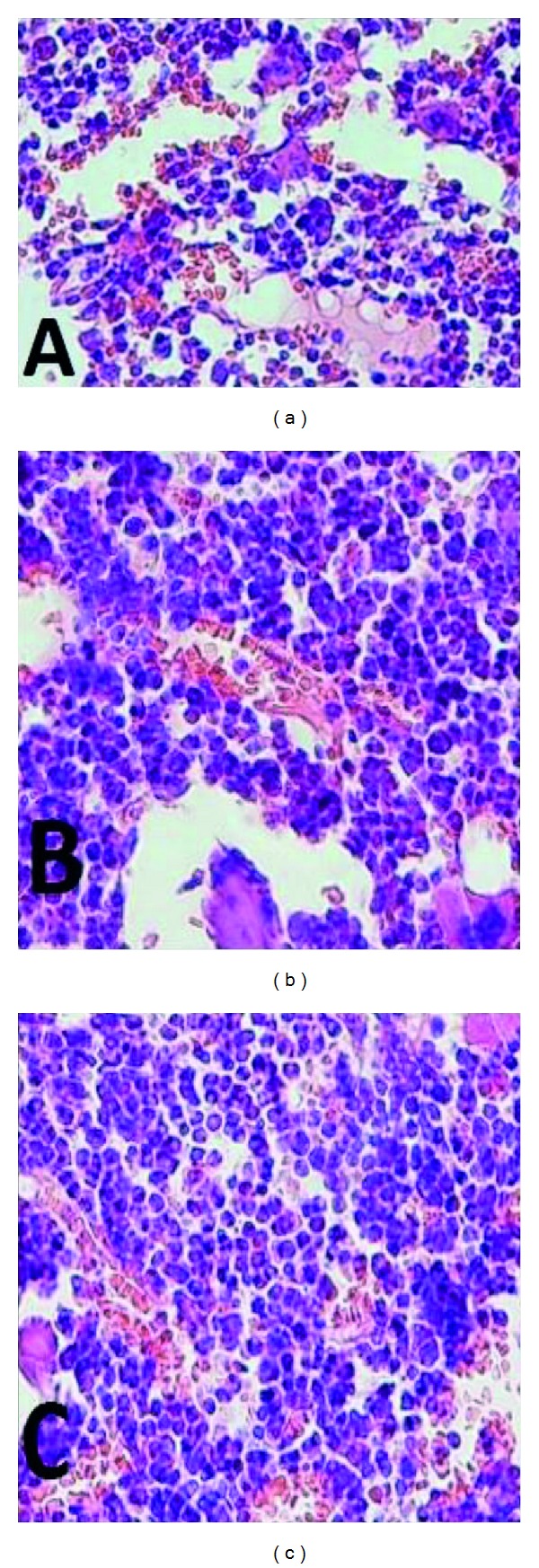
Myeloid hypoplasia in femur 24 hours after CP administration 70 mg/kg; (a) compared with myeloid hyperplasia induced by administration of CCT fruits extract at 100 mg/kg; (b) & 200 mg/kg; (c) for 7 consecutive days before CP treatment. CP-induced myelosuppressive effects (a) and CCT led to marked proliferation and hypercellularity of immature myeloid elements after mice were treated with CP and mitigated the bone marrow suppression ((b) and (c)). Note the relative increase in the proportion of myeloid to erythroid precursors after CCT administration (hematoxylin and eosin-stained paraffin sections).

Histological examination of the bone marrow showed that administration of CP induced myelosuppressive effects (Figure 2(a)). Administration of 100 and 200 mg/kg CCT led to marked proliferation and hypercellularity of immature myeloid elements after mice were treated with CP, as well as mitigated bone marrow suppression (Figures 2(b) and 2(c)).

## 4. Discussion

The results from our study show the ability of CP to induce the formation of micronuclei in PCEs in the bone marrow of mice. The induction of micronuclei is commonly used to assess chromosomal damage [25–27]. There are many reports that hazardous environmental chemicals are capable of inducing genotoxic stress and carcinogenic effects on the mammary gland. These effects are primarily due to the production of free radical species that damage critical macromolecules, such as DNA, to promote chronic diseases including cancer. Although the human body is equipped with self-defense mechanisms, exposure to high levels of dangerous chemicals can lead to mutagenic and carcinogenic events [28]. The cellular and tissues toxicity were observed in the increased therapeutic dose of CP. CP and its metabolites can bind DNA, causing damage that may result in chromosome breaks, micronucleus formation, and cell death [7, 29]. We previously reported CP-induced oxidative stress and genotoxicity in mice bone marrow cells [10, 11]. In our recent study, CP administration induced testicular toxicity and oxidative stress in testis tissues of male mice [30]. The cellular mechanisms by which CP causes testicular injury are poorly understood; however, numerous studies have shown that CP treatment is associated with induction of oxidative stress by the generation of free radicals and ROS [31, 32].

The consumption of vegetables and fruits has been an effective strategy for reducing the genotoxicity and carcinogenicity induced by hazardous chemicals or radiation. The preventive effects of natural products are primarily due to their antioxidant and free radical-scavenging activities [33, 34].

CCT is an important medicinal plant belonging to the family Cucurbitaceae. Several active chemical constituents of CCT plant were recorded. A number of secondary metabolites have previously been reported in this plant including cucurbitacins, flavonoids, caffeic acid derivatives, and terpenoids in addition to flavone glycosides and cucurbitacin glucosides [35, 36]. CCT also contains flavonoids such as quercetin, myricetin, and kaempferol [37]. In our study, the CCT extract scavenged the DPPH free radical in a dose-dependent manner. The maximum antioxidant activity was observed at a concentration of 3 mg/mL that inhibited 95.862% of DPPH free radical (Figure 1), which is totally in agreement with previous studies. In Kumar et al. study, free radical-scavenging effect of CCT fruit extract increased with increasing concentration, and maximum antioxidant activity was observed at 2.5 mg/mL with the percentage inhibition of 88.0 ± 2.7 [38]. Delazar et al. isolated and identified three types of flavonoid, flavone glucosides, and two cucurbitacin glucosides from the methanol extract of the endemic Iranian species CCT fruits. The antioxidant property of these flavonoids was determined by the DPPH assay and showed significant antioxidant properties [35]. We previously reported that medicinal plants and natural products such as flavonoids and phenolic compounds with antioxidant properties and free radical-scavenging mechanism reduced genotoxicity and micronucleus formation in human blood lymphocytes when administrated prior to genotoxic agents [39–42]. We recently reported that *O. vulgare* pretreatment attenuated radiation-induced oxidative stress and the subsequent DNA damage in human blood lymphocyte. The protective effect of *O. vulgare* on DNA could be explained by its ability to increase activity of antioxidant defense system, scavenge the ROS that induce lipid peroxidation as well as peroxidative damage, and quench free radicals induced by internal radiation [33].

CP is a well-known bifunctional alkylating agent widely used in cancer chemotherapy and expresses its genotoxicity when metabolically activated [43, 44]. Normal tissues injury or damage is the major limitation of using CP, which gives rise to numerous side effects; CP treatment also results in the production of ROS, which cause peroxidative damage to vital organs [45]. CP and its metabolites can bind DNA, causing damage that may result in chromosome breaks, Mn formation, and cell death [7, 29]. Medicinal plants have potential preventive properties because of chemical constituents such as phenolic compounds and flavonoids. The biological benefits of these compounds are generally thought to be a result of their antioxidant and free radical-scavenging properties [28]. In a previous study, administration of CCT increased the activity of antioxidant enzymes defense system in liver and kidney of diabetic rats and helped to control the free radical, as the plant is known to be rich in flavonoids and triterpenoids, well-known antioxidants which scavenge the free radicals generated during diabetes [46]. Cucurbitacins, triterpenoid steroids, are one of the major constituents in CCT. Cucurbitacins are efficient antioxidants, and this property lies in their ability to scavenge free radicals such as hydroxyl radical, superoxide anions, and singlet oxygen. Reports also show that cucurbitacins adequately inhibit lipid peroxidation and oxidation [47]. The results of previous studies suggested that CCT contains a free radical-scavenging activity, which could exert a beneficial action against pathological alterations caused by the presence of free radicals and ROS in degenerative diseases [46]. Since ROS are important contributors to tissue injury, inflammation, cancer, and many other ailments, the antioxidant property of compounds probably contributes to the pharmacological and traditional medicinal uses of the CCT [35].

Hence, we evaluated the chemoprotective effects of CCT fruits extract against genotoxicity induced by CP in mouse bone marrow for the first time. CCT extract had dose-dependent protective effects, reduced the frequency of MnPCE induced by CP, and increased proliferation of bone marrow cellularity that was affected by CP. Administration of 100 and 200 mg/kg of CCT to mice prior to the injection of CP reduced the frequency of MnPCE approximately 3.88- and 6.37-fold, respectively. CCT treatment also attenuated the bone marrow suppression, which declined in mice treated with CP. Our study also documented a significant rise in the level of MDA in CP only treated group. This rise in bone marrow MDA levels is a consequence of increased lipid peroxidation. MDA, the product of lipid peroxidation, can interact with DNA, causing strand breaks that in turn develop into chromosomal breaks [48]. CCT showed antilipid peroxidation activity which significantly decreased the levels of MDA. This decrease in lipid peroxidation by CCT might be due to the scavenging of free radicals and ROS. Moreover, CP has a prooxidant nature, and production of oxidative stress after CP administration leads to decrease in the activities of antioxidant enzymes in different tissues of mice and rats [49, 50]. This can induce genotoxicity through the failure of the antioxidant defense mechanisms. Cellular GSH plays an important role in the antioxidant defense system. The ratio of intracellular GSH/GSSG is also often used as an indicator of the cellular redox state, the degree of oxidative stress, and the antioxidant capacity of cells [51]. We demonstrated that CCT pretreatment reduced the CP-induced oxidative stress and increased the GSH/GSSG ratio significantly. The increased GSH and GSH/GSSG level suggests that protection by CCT may be mediated through the modulation of cellular antioxidant levels. These results are in agreement with other studies. Previously, CCT pulp extract possessed a potent antioxidant property against oxidative stress in the RBC's of alloxan induced diabetic rats. Chronic oral administration of CCT pulp extract restored the altered levels of the enzymatic components of the antioxidant system to their normal levels [52].

## 5. Conclusion

Our study provides evidence that CCT pretreatment attenuates CP-induced oxidative stress and the subsequent DNA damage in mice. CCT had potentially protective and anticlastogenic effect against the genotoxicity and Mn formation induced by CP in mice bone marrow cells. Histological examination of bone marrow also showed that CCT extract mitigates myelosuppressive effect of CP and bone marrow suppression. The protective effect of CCT on DNA could be explained by its ability to increase activity of antioxidant defense system, scavenge the ROS that induce lipid peroxidation as well as peroxidative damage, and quench free radicals that induce DNA strand breaks. Therefore, CCT is a good candidate to help defend the body against side effects, particularly genotoxicity of CP-induced oxidative stress condition during chemotherapy.

## Figures and Tables

**Table 1:** Frequency of MnPCE and mitotic activity (% PCE) in bone marrow cells of mice treated with CCT fruits extract and/or CP.

Group	Treatment	% MnPCE^a^	% PCE^a^
1	Control	0.54 ± 0.08	51.32 ± 6.21
2	CP	6.14 ± 0.36^b^	32.48 ± 4.15^b^
3	CCT 10 mg/kg + CP	5.94 ± 0.72^d^	31.28 ± 7.85^d^
4	CCT 50 mg/kg + CP	3.62 ± 0.81^f^	40.62 ± 5.14^f^
5	CCT 100 mg/kg + CP	1.04 ± 0.27^c,e^	48.89 ± 6.78^c,e^
6	CCT 200 mg/kg + CP	0.92 ± 0.06^c,e^	50.32 ± 8.01^c,e^
7	CCT 200 mg/kg	0.47 ± 0.14^e^	51.79 ± 5.46^e^

CCT: *Citrullus colocynthis*; CP: cyclophosphamide; MnPCE: micronucleated polychromatic erythrocyte.

^a^Values are the mean ± standard deviation for each group of 5 mice. ^b^ *P* < 0.0001 compared to the control; ^c^ *P* < 0.0001 compared with the CP treated group; ^f^ *P* < 0.001 compared with the CP treated group. ^d^No significant difference compared to the CP group. ^e^No significant difference compared to the control group.

The data were analyzed with one-way ANOVA and Tukey's HSD test.

**Table 2:** Levels of MDA, GSH, GSSG, and GSH/GSSG ratio in bone marrow of mice after treatment with CCT (200 mg/kg) and/or CP (70 mg/kg).

Groups (mg/kg)	MDA (*µ*mol/g protein)	GSH (*µ*mol/g protein)	GSSG (*µ*mol/g protein)	GSH/GSSG ratio
Control	0.74 ± 0.17	13.64 ± 4.36	3.38 ± 0.43	4.03 ± 1.07
CP	3.07 ± 0.42^b^	3.81 ± 1.47^b^	13.38 ± 3.76^b^	0.28 ± 0.05^b^
CCT	0.71 ± 0.32^c^	14.69 ± 5.16^c^	2.97 ± 0.35^c^	4.94 ± 1.72^c^
CCT + CP	1.32 ± 0.24^d^	12.37 ± 3.84^e^	6.14 ± 3.84^d^	2.01 ± 0.63^d^

CCT: *Citrullus colocynthis*; CP: cyclophosphamide; MDA: lipid peroxidation; GSH: reduced glutathione; GSSG: oxidized glutathione.

^a^Values are the mean ± standard deviation for each group of 5 mice. ^b^ *P* < 0.0001 compared to the control. ^c^No significant difference compared to the control group; ^d^ *P* < 0.001 compared with the CP treated group;^e^ *P* < 0.0001 compared with the CP treated group.

The data were analyzed with one-way ANOVA and Tukey's HSD test.
